# Clinical Implications of p16 Evaluation in a Purposively Sampled Cohort of High-Risk Breast Cancer Phenotypes

**DOI:** 10.3390/ijms27094097

**Published:** 2026-05-03

**Authors:** Sorana Caterina Anton, Alin Horațiu Nedelcu, Carmen Rodica Anton, Ionela Daniela Morariu, Ancuța Lupu, Gabriel Dăscălescu, Alin Ciobîcă, Vasile Valeriu Lupu, Anton Knieling, Dragoș Valentin Crauciuc, Carp Eduard, Mihaela Tirnovanu, Iurie Dondiuc, Ciprian Ilea, Emil Anton

**Affiliations:** 1 Grigore T. Popa University of Medicine and Pharmacy, 700115 Iasi, Romania; sorana.anton@umfiasi.ro (S.C.A.); alin.nedelcu@umfiasi.ro (A.H.N.); ionela.morariu@umfiasi.ro (I.D.M.); ancuta.ignat1@umfiasi.ro (A.L.); vasile.lupu@umfiasi.ro (V.V.L.); anton.knieling@umfiasi.ro (A.K.); dragos.crauciuc@umfiasi.ro (D.V.C.); westfallia@gmail.com (C.E.); mihaela.tirnovanu@umfiasi.ro (M.T.); emil.anton@umfiasi.ro (E.A.); 2 Faculty of Biology, Alexandru Ioan Cuza University of Iasi, 700506 Iasi, Romania; gabidascalescu2001@gmail.com (G.D.); alin.ciobica@uaic.ro (A.C.); 3 Ioan Hăulică Institute, Apollonia University of Iasi, 700511 Iasi, Romania; 4 CENEMED Platform for Interdisciplinary Research, Grigore T. Popa University of Medicine and Pharmacy, 700511 Iasi, Romania; 5 Olga Necrașov Center, Biomedical Group, Romanian Academy, Iasi Branch, Teodor Codrescu 2, 700481 Iasi, Romania; 6 Faculty of Medicine, Nicolae Testemitanu University of Medicine and Pharmacy, 2004 Chisinau, Moldova; iurie_dondiuc@yahoo.com

**Keywords:** p16, invasive breast carcinoma, triple-negative breast carcinoma (TNBC), estrogen receptor (ER), immunohistochemistry, biomarker

## Abstract

The overexpression of cyclin-dependent kinase inhibitor p16 (INK4a) is widely recognized as a surrogate marker for high-risk human papillomavirus (HPV) in anogenital malignancies, but its significance in invasive breast carcinoma is complex and remains frequently debated. While historically investigated as a viral proxy, emerging evidence suggests that elevated p16 levels in breast tissue may instead reflect intrinsic cell-cycle dysregulation and retinoblastoma (Rb) pathway disruption, though direct molecular confirmation is lacking in this area of research. This study aims to evaluate the role of p16 as an indicator of tumor aggressiveness for high-risk phenotypes. We conducted a retrospective study of 100 female patients with invasive breast carcinoma. Employing a purposive sampling strategy rather than a consecutive series, we analyzed a targeted cohort consisting predominantly of triple-negative breast cancer (TNBC) and high-grade tumors to evaluate biomarker patterns specifically in advanced disease contexts. Immunohistochemical assessment was performed using a standardized cumulative nuclear and cytoplasmic scoring system, with expression thresholds defined by receiver operating characteristic (ROC) curve analysis optimized for histological grade. p16 overexpression was a predominant characteristic of these aggressive tumors and was identified in 68% of cases. Statistical evaluation revealed a robust and significant correlation between p16 overexpression and the triple-negative molecular subtype, as well as a marked inverse relationship with estrogen receptor (ER) status. Although p16 levels were frequently associated with specific aggressive phenotypes, no statistically significant difference in overall survival was observed between expression groups, a finding attributable to the uniformly high-risk nature of the selected cohort. This study suggests an association between p16 expression levels and aggressive tumor features, although the study design limits causal inferences. A non-significant trend towards p16 overexpression was observed in ductal carcinomas compared to lobular subtypes, while high p16 expression was noted exclusively in G3 tumors within this selected cohort, a finding influenced by the purposive sampling strategy and the ROC-based cutoff definition. Tumor necrosis was more prevalent in p16-overexpressing tumors. Furthermore, p16 levels showed a strong inverse relationship with estrogen receptor (ER) status, as they were significantly elevated in ER-negative and triple-negative tumors compared to luminal phenotypes.

## 1. Introduction

Breast carcinoma remains a complex oncological challenge despite significant research advancements, necessitating the continuous identification of novel biomarkers to elucidate its heterogeneous progression pathways and clinical behavior [1].

Breast carcinogenesis is primarily driven by genetic and epigenetic alterations. In aggressive phenotypes such as triple-negative breast cancer (TNBC), frequent disruption of the retinoblastoma (Rb) pathway leads to compensatory upregulation of p16, reflecting a collapse of the CDK4/6-Rb cell-cycle checkpoint. Understanding this intrinsic pathway is crucial, as it identifies tumors with high proliferative capacity and distinct biological behavior. Although human papillomavirus (HPV) has been historically investigated, its direct etiological role in breast cancer remains debated [2,3,4,5,6,7]. In breast tissue, p16 overexpression often arises independently of viral presence. This marker has been widely explored as a surrogate for HPV-driven carcinogenesis in anogenital malignancies [8]. In those viral contexts, the E7 oncoprotein degrades Rb, triggering compensatory p16 upregulation [9]. However, in breast carcinoma, p16 elevation typically stems from intrinsic mechanisms. These include Rb1 pathway disruption and cyclin D1 amplification [10]. Mechanistically, p16 inhibits CDK4/6-cyclin D complexes, preventing Rb phosphorylation and subsequent E2F release [11]. Therefore, elevated p16 in breast tumors may reflect a state of oncogene-induced senescence or a “broken brake” phenomenon in highly proliferative tumors rather than purely suppressive activity [12].

This biological complexity is reflected in the biomarker’s subtype-specific prevalence, as p16 overexpression is documented in 45–80% of basal-like and TNBC cases compared to less than 15% in luminal A tumors [13,14]. Paradoxically, the prognostic implications of p16 are multifaceted. While some studies link p16 negativity to aggressive phenotypes [13], meta-analyses in other gynecological malignancies, such as vulvar cancer, have associated p16 overexpression with lower FIGO stages, absence of lymph node metastasis, and improved overall survival [15]. Furthermore, in specific contexts, such as gastric-type cervical carcinoma, aberrant stromal overexpression of p16 has been linked to lymphovascular invasion, suggesting a role in clinically aggressive behavior [16]. The interpretation of these patterns remains challenging due to the dual nuclear and cytoplasmic localization of the protein, which necessitates standardized scoring protocols to ensure reproducibility [17].

Current evidence suggests that the prognostic utility of p16 in breast oncology may supersede its association with viral etiology, particularly in stratifying progression risk in TNBC and determining the invasion potential of ductal carcinoma in situ (DCIS) [18,19]. Therefore, this study aims to explore, in detail, the dual role of p16 protein within the specific context of invasive breast carcinoma. By employing immunohistochemistry alongside rigorous clinical and molecular analyses, we sought to elucidate whether p16 serves merely as an indirect marker of high-risk HPV infection or, more critically, as an independent indicator of cell-cycle deregulation, evidenced by protein accumulation, which influences tissue-level aggressiveness and disease evolution. Through this approach, we aim to address existing knowledge gaps regarding viral versus intrinsic carcinogenesis in breast tissue and provide a nuanced perspective to support therapeutic decision-making and long-term prognostic assessment.

## 2. Results

### 2.1. Clinicopathological Characteristics of the Study Cohort

The study cohort included 100 female patients diagnosed with invasive breast carcinoma, with ages ranging from 38 to 90 years. The majority of the cohort (72.0%) was aged between 51 and 80 years. Stratified analyses revealed that the 71–80 age group was the largest subgroup (30.0%); within this demographic, low p16 expression was the predominant pattern (43.8% of low expressors). Conversely, high p16 expression was more frequently distributed across the 51–60 (26.5%) and 71–80 (23.5%) age intervals. Despite these observational trends, statistical analyses indicated no significant association between patient age and p16 expression levels (*p* = 0.338). A comprehensive univariate analysis of all clinicopathological variables relative to p16 status is presented in Table 1.

Histologically, the cohort consisted predominantly of invasive ductal carcinoma (IDC), which accounted for 84% of cases, followed by invasive lobular carcinoma (ILC) at 11% and other specific variants at 5%. A trend towards elevated p16 expression was observed in ductal carcinomas (89.7% of high expressors) compared to lobular subtypes (7.4% of high expressors), although this difference approached but did not reach statistical significance (*p* = 0.075; Cramer’s V = 0.228).

Regarding tumor grade, the cohort was characterized by a high prevalence of high-grade malignancy, with 98% of cases classified as G3 and only 2% classified as G2. While low p16 expression was observed in G2 and G3 tumors, high p16 expression was exclusively restricted to G3 tumors (100% of high-p16 cases were G3). However, because our purposive sampling resulted in only two G2 tumors within the entire cohort, this binary distribution is strictly descriptive. The extremely small sample size of the G2 subgroup precludes any robust statistical validation of an exclusive association with high histological grade, and this trend should be interpreted with appropriate caution.

### 2.2. Immunohistochemical Expression Patterns

**Figure 1:**
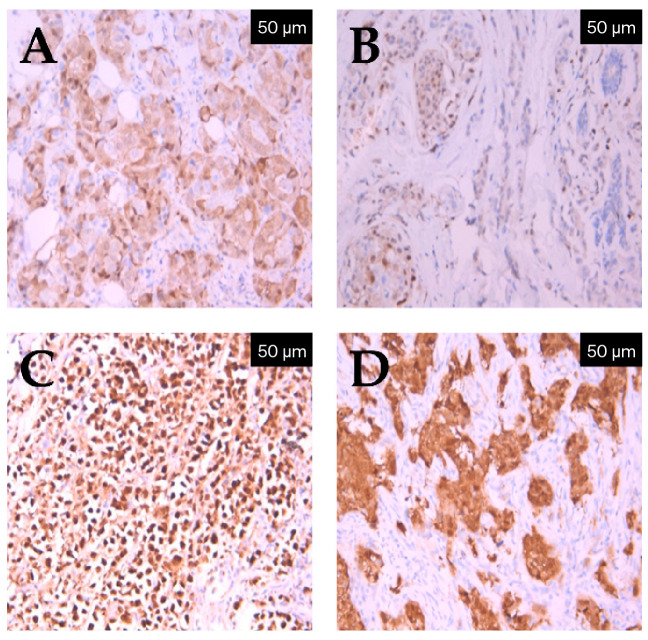
Representative immunohistochemical expression levels of p16 (IHC, anti-p16 monoclonal antibody, and clone 6H12) in invasive breast carcinoma. (**A**) Low p16 expression in invasive ductal carcinoma (IHC, ×20); (**B**) low p16 expression in invasive lobular carcinoma (IHC, ×20); (**C**) high p16 expression showing diffuse nuclear and cytoplasmic staining in invasive lobular carcinoma (IHC, ×20); (**D**) high p16 expression in a high-grade triple-negative ductal carcinoma (IHC, ×20).

To substantiate scoring reproducibility and illustrate the diverse subcellular localization of the protein, representative immunohistochemical fields corresponding to the various histological subtypes and expression levels were analyzed. As illustrated in Figure 1, p16 staining patterns ranged from focal or absent expression to diffuse, strong nuclear, and cytoplasmic positivity, particularly in high-grade ductal carcinomas.

### 2.3. Correlation with Tumor Microenvironment and Invasion

We assessed markers of local aggressiveness to determine whether p16 overexpression correlated with specific invasion patterns. Lymphovascular invasion (LVI) was identified in 17% of the total cohort. The frequency of LVI was comparable between the p16-low (18.8%) and p16-high (16.2%) groups, yielding no statistically significant correlation (*p* = 0.749). Similarly, perineural invasion (PNI) was a rare event observed in only 5% of cases, with no significant variation based on p16 status (*p* = 0.654).

Assessments of the tumor microenvironment showed that peritumoral mononuclear inflammatory infiltrates were present in varying densities (absent: 29%; mild: 30%; moderate: 29%; rich: 12%), but their distribution did not correlate with p16 expression levels (*p* = 0.884). Tumor necrosis was present in 38% of cases. Tumor necrosis was more frequently observed in p16-high tumors (42.6%) compared to p16-low tumors (28.1%). While this finding is consistent with the aggressive nature of p16-overexpressing phenotypes, the difference did not reach statistical significance (*p* = 0.163).

### 2.4. Associations with Hormone Receptors and HER2 Status

A key characteristic of this cohort was the low prevalence of hormone receptor positivity. Estrogen receptor (ER) positivity was identified in only 10% of tumors. Statistical analysis revealed a significant inverse correlation (*p* = 0.011; Cramer’s V = 0.272): ER positivity was more frequent in the p16-low group (21% of low cases) compared to the p16-high group (4.4% of high cases), indicating that p16 overexpression is significantly associated with ER-negative disease (Table 1).

Progesterone Receptor (PR) positivity was similarly low (8%) and showed no statistically significant difference between expression groups (*p* = 0.264). HER2 overexpression was detected in 5% of the cohort, with no significant stratification by p16 status (*p* = 0.324). Notably, the Ki-67 proliferative index exceeded 50% in all analyzed cases, confirming the uniform high-proliferative status of the study population.

### 2.5. p16 Expression and Molecular Subtypes

The molecular classification distribution was as follows: 88% TNBC, 10% Luminal B, and 2% HER2. A statistically significant association was identified between p16 overexpression and the TNBC subtype (*p* = 0.012; Cramer’s V = 0.280). Specifically, 94.1% of all tumors with p16 overexpression were classified as triple-negative compared to 75% of tumors with low p16 expression. These findings identify p16 overexpression as a predominant feature of the triple-negative phenotype in this cohort. A comprehensive univariate analysis of all clinicopathological variables is presented in Table 1.

### 2.6. Survival Analysis

**Figure 2:**
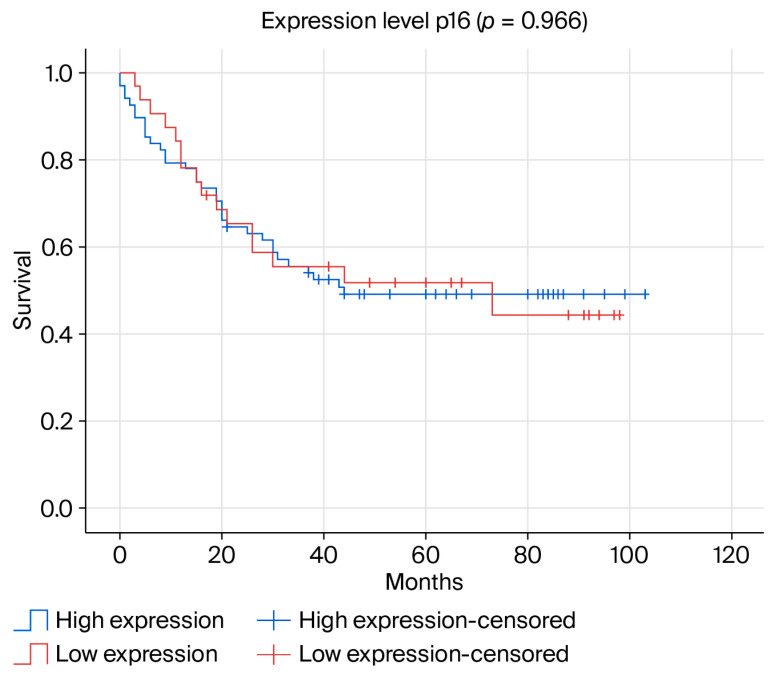
Kaplan–Meier overall survival curve comparing low vs. high p16 expression groups.

Kaplan–Meier survival analysis was performed to evaluate the prognostic impact of p16 expression. In this specific cohort, characterized by uniformly aggressive features (98% G3, high Ki-67), no statistical difference in overall survival (OS) was observed between the p16-low and p16-high groups (*p* = 0.966, log-rank test), as depicted in Figure 2.

## 3. Discussion

This study presents a comprehensive immunohistochemical evaluation of p16 protein expression within a specifically selected cohort of 100 invasive breast carcinomas. We investigated the significance of p16 in a high-risk context that extends beyond its traditional, and often debated, role as a viral surrogate. Our central finding indicates p16 overexpression in 68% of cases, with a distinct predilection for TNBC. This aligns with the hypothesis that p16 upregulation in breast malignancy reflects intrinsic cell-cycle dysregulation. It likely represents a “senescence escape” driven by Rb pathway dysfunction, rather than acting solely as a marker of HPV infection.

Demographic analyses revealed that while p16 overexpression was distributed across various age groups, a notable accumulation was observed in post-menopausal women (51–80 years). Although this association did not reach statistical significance (*p* = 0.338), the elevated p16 trend in relatively younger post-menopausal subsets echoes the findings reported by Mohammadizadeh and Nasri, potentially signaling the early onset of aggressive phenotypes in this demographic [20]. Furthermore, Chen et al. demonstrated that elevated p16 in women aged 50–60 predicted higher proliferation indices, reinforcing our inference of an active cell-cycle profile in this age group [21].

Regarding histological characteristics, invasive ductal carcinoma comprised the vast majority of our cases (84%), and high p16 expression was more frequently observed in this subtype (89.7%) compared to lobular carcinomas. However, as this variation only indicated an observational trend (*p* = 0.075) and did not reach statistical significance, likely due to the limited number of lobular carcinomas in our sample, this specific distribution should be interpreted cautiously. This tendency supports the observations of Rezaei et al. and Milde-Langosch et al., who documented preferential p16 upregulation in ductal malignancies [22,23]. Rezaei et al. further noted that diffuse p16 staining in ductal carcinoma correlated with significantly reduced disease-free survival [22].

Our descriptive data showed that every high-p16 tumor in our cohort was classified as Grade 3. However, this finding must be interpreted with caution. First, histological grade was utilized as the reference variable in our exploratory ROC analysis to define the p16 cutoff, introducing a degree of methodological circularity. Second, the purposive sampling strategy resulted in only two G2 tumors within the entire cohort, which precludes any robust statistical validation of an exclusive association with high histological grade. Thus, while our observation is consistent with previous reports linking elevated p16 to poorly differentiated phenotypes [24], it should not be construed as an independent association without validation in more balanced, unselected datasets.

Contrary to the hypothesis linking p16 to specific patterns of local infiltration, we found no significant correlation between expression levels and lymphovascular (LVI) or perineural invasion (PNI). This aligns with recent data from Davidson et al. and Johnson et al., who reported that p16 status did not independently predict LVI in squamous cell carcinomas, suggesting that p16 reflects tumor-intrinsic proliferative capacity rather than the mechanical ability to infiltrate vascular structures [25,26]. Similarly, the distribution of mononuclear inflammatory infiltrates did not vary significantly by p16 status, a finding consistent with De Wispelaere et al., indicating that p16 overexpression does not, on its own, elicit a distinct host immune response in this setting [27]. Tumor necrosis was more frequent in p16-high tumors (42.6%) than in low-expressing ones (28.1%). While this trend did not exhibit statistical significance (*p* = 0.163), it aligns with reports linking the p16/cyclin D1 axis to HIF-1α upregulation. This suggests a metabolic phenotype where rapid neoplastic growth outstrips vascular supply [28,29].

The most clinically relevant finding of our study is the robust inverse correlation with ER status (*p* = 0.011) and the strong association with the TNBC molecular subtype (*p* = 0.012). In our cohort, 94.1% of high-p16 tumors were triple-negative. This distribution provides strong evidence for the “Rb-p16 inverse relationship” described in the literature. As noted by Lebok et al. and Arima et al., p16 loss is associated with stemness and resistance in some ER-negative lines, but its overexpression, as seen here, signals a different mechanism [10,30]. Ni et al. suggested that p16 status could predict responses to CDK4/6 inhibitors even in HER2+ disease, hinting at its broad utility in cell-cycle stratification [31]. Crucially, our results align with the very recent findings of Lee et al. (2025), who demonstrated that diffuse p16 patterns in TNBC frequently co-occur with genomic signatures of pRb loss [32]. Similarly, Yoon et al. found p16 co-expression with basal markers such as SOX10 [33]. Our data are consistent with a specific mechanistic model. In the absence of functional Rb, the negative feedback loop is disrupted. This disruption results in a compensatory and marked overexpression of p16. Thus, p16 levels could serve as a surrogate “readout” of the CDK4/6-Rb pathway collapse in basal-like tumors [34,35].

The lack of a significant difference in OS (*p* = 0.966) reflects our study design. The cohort was enriched for aggressive, high-grade tumors, meaning the “baseline” aggressiveness level was uniformly elevated. This likely masked prognostic separation visible in more heterogeneous populations. In such high-risk groups, p16 serves more as a phenotypic descriptor than an independent prognostic factor for mortality [13,36].

A critical consideration when interpreting these findings is the purposively selected nature of our study cohort. Because we deliberately structured the study population to predominantly include TNBC (88%) and histological Grade 3 (98%) carcinomas in order to investigate high-risk phenotypes, our results cannot be directly generalized to the broader, unselected breast cancer population. Furthermore, due to this intentional selection framework, p16 cannot be evaluated in this study as a *de novo* predictor of tumor aggressiveness, as highly aggressive features were foundational inclusion criteria. Rather, our data delineates the specific distribution and biological behavior of p16 once the high-risk phenotype is already established.

While this study offers valuable insights into the behavior of p16 in aggressive breast cancer, we acknowledge certain limitations inherent to the retrospective design. The primary constraint, as noted in similar investigations, is the reliance on immunohistochemistry without concurrent molecular genotyping for HPV DNA or RNA or direct protein-level validation of the Rb pathway (e.g., RB1 status, CDK4/6 activity, or genomic profiling). This restricts our ability to definitively rule out viral etiology or to conclusively prove intrinsic RB pathway disruption. While the robust correlation with intrinsic TNBC markers strongly points towards a non-viral mechanism, our mechanistic interpretations regarding viral versus intrinsic pathways remain hypothesis-generating and require future experimental validation. Based on these findings, several strategic directions for future research emerge. First, the assessment of p16 expression by IHC could become a valuable therapeutic stratification tool in clinical trials investigating CDK4/6 inhibitors, especially in tumors with Rb dysfunction or hormone refractoriness [31,37]. Such applications remain speculative at this stage and will require prospective validation in appropriately designed clinical trials. Second, in-depth mechanistic studies are needed to elucidate the epigenetic interaction between KDM6B and p16, especially in the context of TNBC, a subtype with poor prognosis and limited therapeutic options [9,38]. A promising approach is the integration of p16 into complex biomarker panels, including Rb protein, viral genetic material, and transcriptomic profiles, to increase specificity and sensitivity in tumor diagnosis and prognosis [39]. Finally, the prospective validation of these findings requires the initiation of large-scale multicenter studies, including comprehensive genomic and immune profiling, to confirm the prognostic reliability and predictive capacity of p16 in various oncological contexts [40].

Additionally, while two senior pathologists independently evaluated p16 expression and discordant cases were resolved by consensus, a formal inter-observer reproducibility metric (e.g., Cohen’s kappa) was not calculated. This limits the quantitative assessment of scoring consistency, although the consensus-based approach mitigated variability in the final classification.

Furthermore, the limited sample size within several clinically relevant subgroups (e.g., G2, HER2-positive, PR-positive, and lobular histology) reduced statistical power for detecting associations that might reach significance in larger cohorts. Consequently, non-significant trends reported for variables such as age, histological type, and tumor necrosis should be viewed as hypothesis-generating and warrant validation in larger, more balanced studies.

## 4. Materials and Methods

### 4.1. Study Design and Patient Selection

This study was designed as a retrospective observational cohort study focused on evaluating the clinicopathological and prognostic significance of p16 expression in high-risk breast malignancy. The study population comprised 100 female patients diagnosed with primary invasive breast carcinoma (IBC) who underwent surgical resection and adjuvant therapy at the Regional Institute of Oncology Iasi and the “Cuza Voda” Clinical Hospital of Obstetrics and Gynecology, Iasi, between January 2015 and December 2024. Eligible cases were identified via a systematic review of oncology charts, operative reports, and pathology archives. Rather than an a priori power calculation, a convenience sample of exactly 100 cases was established. This final cohort size was pragmatically determined by our rigorous inclusion and exclusion criteria; specifically, only cases with completely intact clinicopathological records, full molecular receptor status, and sufficient uncompromised FFPE tissue blocks, without prior neoadjuvant therapy, were selected to ensure absolute data reliability. It is important to note that the small subgroup sizes for certain variables (e.g., G2 tumors, *n* = 2; HER2-positive, *n* = 5; PR-positive, *n* = 8) limit the statistical power for secondary analyses involving these subgroups, and findings related to these categories should be interpreted as descriptive only. Inclusion criteria required the availability of sufficient formalin-fixed, paraffin-embedded (FFPE) tumor tissue for immunohistochemical processing and complete clinicopathological records, including age at diagnosis, histological type, grade, lymphovascular invasion (LVI) status, perineural invasion (PNI), tumor necrosis, and full molecular receptor status (ER, PR, and HER2). Patients with incomplete clinical data, insufficient biological material, or those who received neoadjuvant chemotherapy prior to tissue sampling were excluded to avoid alterations in baseline biomarker expression. It is important to note that this study did not utilize a consecutive case series; rather, a purposive sampling methodology was applied. Given the focus on high-risk phenotypes, the selection criteria were deliberately structured to assemble a cohort with a high prevalence of triple-negative and high-grade tumors. This approach ensured adequate statistical power for rigorously evaluating p16 behavior within these specific, highly aggressive clinical contexts.

### 4.2. Immunohistochemical Procedures

Immunohistochemical (IHC) analysis was performed on 4 µm thick sections derived from representative FFPE tissue blocks. Staining was automated using the BOND platform (BOND-MAX and BOND-III systems, Leica Biosystems, Nußloch, Germany), utilizing a ready-to-use monoclonal anti-p16 antibody (clone 6H12, Catalog No. PA0016-U). This antibody targets the full-length human p16 protein. As it is a commercially optimized, ready-to-use (RTU) formulation (Catalog No. PA0016-U; Leica Biosystems, Newcastle upon Tyne, UK), it was applied strictly according to the manufacturer’s protocol without any manual dilution or pre-titration, ensuring standardized inter-assay staining. The protocol included deparaffinization and heat-induced epitope retrieval using Epitope Retrieval Solution 2 (pH 9.0; Leica Biosystems, Newcastle upon Tyne, UK), followed by incubation with the primary antibody. Signal detection was achieved using the BOND Polymer Refine Detection system (DS9800) and BOND-PRIME Polymer DAB Detection (DS9284) (Leica Biosystems, Newcastle upon Tyne, UK), with 3,3′-diaminobenzidine (DAB) as the chromogen. Appropriate positive and negative controls were included in each run to validate staining specificity. Microscope evaluation and image acquisition were performed using a Leica DM3000 LED light microscope (Leica Biosystems, Nußloch, Germany).

### 4.3. Scoring System and Definition of Overexpression

To assess p16 expression levels, a combined semi-quantitative scoring system was employed. Two senior pathologists independently evaluated the slides, assessing both nuclear and cytoplasmic staining intensities on a scale from 0 to 3 (0 = negative; 1 = weak; 2 = moderate; 3 = strong). A final composite score was calculated by summing nuclear and cytoplasmic values, yielding a total range of 0 to 6. While a formal inter-observer reproducibility metric (e.g., Cohen’s kappa) was not retrospectively calculated, rigorous scoring consistency was maintained procedurally. The two senior pathologists evaluated all cases independently, and any discordant evaluations were systematically resolved through dual-head microscope consensus review to establish the final score. To establish a biologically relevant threshold for p16 overexpression, an exploratory receiver operating characteristic (ROC) curve analysis was performed using histological grade as a proxy for extreme aggressiveness. The analysis yielded an Area Under the Curve (AUC) of 0.770 (*p* = 0.001). The optimal cutoff was determined using Youden’s index (J = 0.6939). Consequently, a cutoff score of >2 was defined as “high p16 expression” (overexpression), while scores of ≤2 were categorized as “low p16 expression”. This specific threshold was deliberately selected to maximize sensitivity (100%) for identifying high-grade lesions, accepting the corresponding trade-off in specificity (69.39%) to ensure that no highly aggressive phenotypes were missed.

### 4.4. Clinicopathological Assessment and Molecular Classification

Tumors were classified histologically as invasive ductal carcinoma (IDC), invasive lobular carcinoma (ILC), or other specific subtypes. Histological grading was determined according to the Nottingham Grading System. Molecular subtypes were defined based on surrogate immunohistochemical markers as follows: Luminal B (ER-positive and/or PR-positive, with HER2-positive status or high Ki-67); HER2-enriched (HER2 overexpression/amplification, ER/PR-negative); and triple-negative (ER-, PR-, and HER2-negative). Due to the high-risk nature of the selected cohort, where the Ki-67 proliferative index exceeded 50% across all cases, the Luminal A subtype was naturally excluded from the classification scheme. HER2 status was evaluated according to standard ASCO/CAP guidelines, with equivocal IHC cases confirmed via silver in situ hybridization (SISH).

### 4.5. Statistical Analysis

Data analysis was executed using IBM SPSS Statistics version 31.0.1.0 (IBM Corp., Armonk, NY, USA). Categorical variables were compared using the Pearson Chi-square test, while Fisher’s exact test was strictly applied whenever expected cell frequencies fell below 5, ensuring the robustness of comparisons for small subgroups. To quantify the clinical relevance of these associations beyond mere statistical significance, effect sizes were estimated using Cramer’s V, where values of >0.25 were interpreted as indicative of a strong practical effect in this specific oncological context. Overall survival (OS) was defined as the time interval from the date of histopathological diagnosis to the date of death from any cause. Patients who were alive at the time of the last follow-up were censored. Survival probabilities were estimated using the Kaplan–Meier method, and differences between groups were assessed via the log-rank test. We relied primarily on univariate modeling. Our purposive sampling strategy deliberately enriched the cohort with extreme phenotypes. This resulted in severe categorical imbalances, such as 98% Grade 3 and 88% TNBC. In such skewed, low-variance contexts, attempting multivariate logistic or Cox regression is mathematically contraindicated. These models carry a high risk of quasi-complete data separation and severe overfitting. To maintain absolute statistical integrity, multivariate adjustment was deemed unfeasible. Our conclusions are drawn strictly from robust univariate associations. A *p*-value < 0.05 was considered statistically significant.

### 4.6. Ethical Considerations

This study was conducted in strict accordance with the ethical principles outlined in the Declaration of Helsinki (2013 revision). The research protocol received formal approval from the Ethics Committees of the Regional Institute of Oncology Iasi (Approval No. 925/20.10.2021) and the “Cuza Voda” Clinical Hospital of Obstetrics and Gynecology (Approval No. 379/17.01.2024). Given the retrospective nature of this study, which exclusively involved archival biological samples and anonymized data, the requirement for individual informed consent was waived in accordance with national legislation and institutional regulation. All patient data were processed in compliance with the General Data Protection Regulation (GDPR) (EU) 2016/679, ensuring complete confidentiality and anonymity of subjects.

## 5. Conclusions

The findings of this study highlight the multifaceted role of the p16 protein as a biomarker that may extend beyond its classical association with HPV-driven tumorigenesis. The high prevalence of p16 expression observed in our cohort is consistent with intrinsic cell-cycle dysregulation, such as CDK4/6-Rb pathway disruption, although this interpretation remains hypothesis-generating in the absence of direct molecular validation. Further targeted molecular studies are required to definitively confirm these specific pathway alterations. Although p16 status did not independently predict overall survival in this uniformly aggressive population, its robust correlation with the triple-negative phenotype and its inverse relationship with ER and PR reinforce its utility as a complementary biomarker for stratifying biological aggressiveness. Consequently, we propose that p16 immunohistochemistry should be valued as a practical, cost-effective tool for identifying cell-cycle-active phenotypes, potentially guiding the use of targeted therapies in non-luminal breast cancer. Future research should aim to integrate p16 into comprehensive molecular panels to facilitate a more precise, personalized approach to cancer diagnosis and treatment.

## Figures and Tables

**Table 1:** Univariate analysis of p16 expression and clinicopathological parameters.

Clinicopathologic Parameters		*n*	p16 Expression		*p*-Value	Cramer’s V
			Low	High		
Age	31–40	5	1 (1%)	4 (4%)	0.338	0.245
	41–50	10	2 (2%)	8 (8%)		
	51–60	22	4 (4%)	18 (18%)		
	61–70	20	7 (7%)	13 (13%)		
	71–80	30	14 (14%)	16 (16%)		
	81–90	13	4 (4%)	9 (9%)		
Histological type	Invasive ductal carcinoma	84	23 (23%)	61 (61%)	0.075	0.228
	Invasive lobular carcinoma	11	6 (6%)	5 (5%)		
	Others	5	3 (3%)	2 (2%)		
Histological grade	G2	2	2 (2%)	0 (0%)	0.100	0.208
	G3	98	30 (30%)	68 (68%)		
Lymphovascular invasion	Absent	83	26 (26%)	57 (57%)	0.749	0.032
	Present	17	6 (6%)	11 (11%)		
Perineural invasion	Absent	95	30 (30%)	65 (65%)	0.654	0.039
	Present	5	2 (2%)	3 (3%)		
Mononuclear inflammatory infiltrate	Absent	29	9 (9%)	20 (20%)	0.884	0.090
	Mild	29	11 (11%)	18 (18%)		
	Moderate	30	9 (9%)	21 (21%)		
	Rich	12	3 (3%)	9 (9%)		
Tumor necrosis	Absent	62	23 (23%)	39 (39%)	0.163	0.140
	Present	38	9 (9%)	29 (29%)		
Estrogen receptor status	Absent	90	25 (25%)	65 (65%)	0.011	0.272
	Present	10	7 (7%)	3 (3%)		
Progesterone receptor status	Absent	92	28 (28%)	64 (64%)	0.264	0.114
	Present	8	4 (4%)	4 (4%)		
HER2 status	Absent	95	29 (29%)	66 (66%)	0.324	0.138
	Present	5	3 (3%)	2 (2%)		
Molecular subtypes	Luminal B	10	7 (7%)	3 (3%)	0.012	0.280
	HER2-positive	2	1 (1%)	1 (1%)		
	Triple-negative	88	24 (24%)	64 (64%)		

## Data Availability

The original contributions presented in this study are included in the article. Further inquiries can be directed to the corresponding author.
